# *Oestrus ovis* external ophtalmomyiasis: a case report in Burgundy France

**DOI:** 10.1186/s12886-018-1003-z

**Published:** 2018-12-22

**Authors:** Louise Basmaciyan, Pierre-Henry Gabrielle, Stéphane Valot, Marc Sautour, Jean-Christophe Buisson, Catherine Creuzot-Garcher, Frédéric Dalle

**Affiliations:** 1 Laboratoire de Parasitologie-Mycologie, Plateau Technique de Biologie, 2 rue A. Ducoudray, 37013, 21070 Dijon Cedex, BP France; 2 UMR PAM Univ Bourgogne Franche-Comté - AgroSup Dijon - Equipe Vin, Aliment, Microbiologie, Stress, VALMIS Team, Bâtiment B3, UFR Sciences de Santé, 2 rue Angélique Ducoudray, 37013 – 21070 Dijon Cedex, BP France; 3 Department of Ophthalmology, University Hospital, F-21000 Dijon, France

**Keywords:** Ophtalmomyiasis, Oestrus ovis, Burgundy

## Abstract

**Background:**

External ophtalmomyiasis (EOM) is a zoonosis related to the presence of *Oestrus ovis* larvae at the ocular level in small ruminants (i.e. ovine, caprine). In humans, EOM is a rare cosmopolitan disorder, mostly described in warm and dry rural areas in patients living close to livestock areas. In metropolitan France (excluding Corsica), EOM is an exceptional disease with less than 25 cases recorded since 1917.

**Case presentation:**

We report a case of EOM in a 19-years old man in the last week of September 2016 in Burgundy.

**Conclusion:**

The diagnosis of an EOM in Burgundy, a French region described as cold and humid, is surprising and could be due to a more marked climatic warming during the vegetative season in Burgundy resulting in the implantation of Diptera of the genus *Oestrus* sp. in this region.

**Electronic supplementary material:**

The online version of this article (10.1186/s12886-018-1003-z) contains supplementary material, which is available to authorized users.

## Background

Ophtalmomyiasis (OM) was introduced in 1840 by Frederik William Hope as a zoonosis defined by the infestation of the ocular apparatus with larvae of Diptera mostly belonging to the families of *Oestridae*, *Calliphoridae* and *Sarcophagidae* [1]. In France, human OM is essentially related to *Oestrus ovis,* a non-picking fly belonging to the family of *Oestridae* [1].

*Oestrus ovis* exerts a strict parasitism of the nasal cavities of small sheep and goat ruminants [2]. The viviparous females of *Oestrus ovis* deposit first-stage larvae (L1) directly in the nasal orifices of sheep and goats. L1 actively penetrate through the nasal orifices and colonize the cornets and septum where they will develop. Once located at the ethmoid level, L1 molt to stage 2 larvae (L2). L2 further ascend from the nasal cavity to the frontal sinuses where they molt to stage 3 larvae (L3). Thereafter, L3 are expelled from the nasal cavity of the host by sneezing via the nasal mucus that subsequently contaminate the soils. Then, L3 turn into a pupa in 12–24 h. Finally, when the external conditions are favorable, the pupa molt into an adult fly in 30 to 34 days [2]. Accidentally, L1 larvae can be deposited on or into the ocular cavities of humans and be responsible for benign (external ophtalmomyiasis (EOM)) or severe (internal ophtalmomyiasis (IOM)) ocular lesions (Additional file 1: Figure S1).

Human EOM occurs usually during summer and early-autumn and is mostly described in rural areas in patients living near livestock areas [3, 4]. In metropolitan France (excluding Corsica), although the number of cases is probably underestimated, EOM is an uncommon disease with less than 25 cases recorded over the last hundred years [5–11]. We report here a case of EOM in a young farm worker in the last week of September 2016 in Burgundy.

## Case presentation

The patient is a 19-year-old man, a seasonal agricultural worker daily in contact with sheep, living in Burgundy and with no history of travel neither abroad nor in the south of France during the previous months. At the end of September 2016, the patient performed farm work in contact with sheep when he had an ocular traumatism caused by a fly. Three hours after the ocular traumatism, the patient complained of a painful right eye discomfort, with sensation of moving foreign. Upon arrival at the department of ophthalmic emergency of the University Hospital Center of Dijon within hours of the onset of the first symptoms, the clinical examination showed a red and irritated conjunctiva in the right eye with the observation of mobile and translucent larvae in the conjunctival fornix. The rest of the ophthalmologic examination was normal. Eight larvae were extracted using Bonn hook forceps under local anesthesia. All larvae were sent to the Parasitology-Mycology Laboratory of the University Hospital Center of Dijon for identification.

**Fig. 1:**
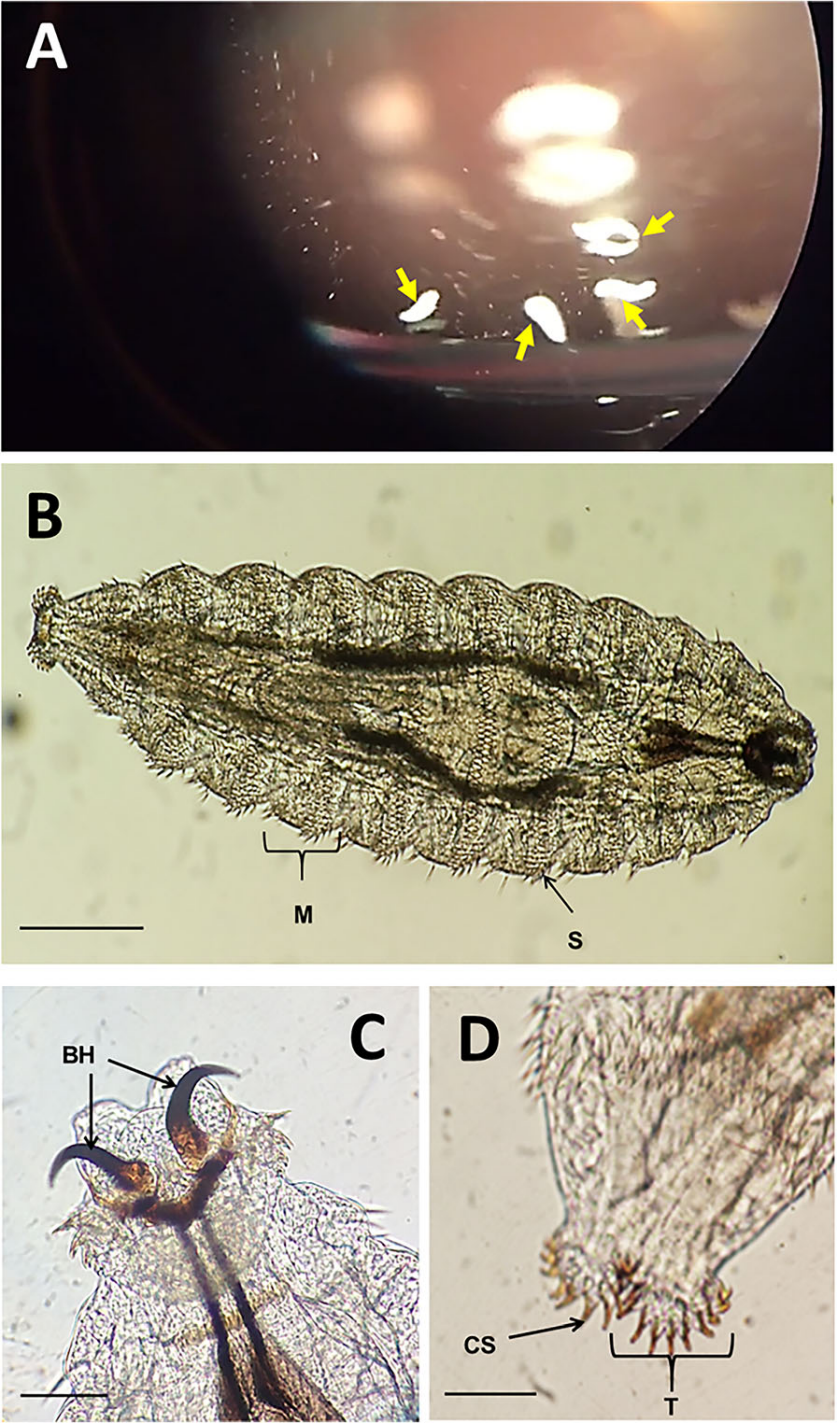
Observation of *Oestrus ovis* larvae (**a**) Observation of translucent larvae of Oestrus ovis in the conjunctival fornix (Yellow arrow: *Oestrus ovis* larvae in the conjunctival fornix). (**b**-**d**) Microscopic observation of *Oestrus ovis* larvae: (**b**) *Oestrus ovis* larvae L1 (× 100) (M = Metamer, S: Spine, Scale bar = 200 μm); (**c**) Cephalic end of *Oestrus ovis* larvae L1 (× 400) (BH: Buccal Hooks; Scale bar = 50 μm); (**d**) Posterior end of the larva L1 of *Oestrus ovis* (× 400) (T: Tubercles, ES: Curved Spine, Scale bar = 50 μm)

The parasitological diagnosis allowed the identification of stage 1 *Oestrus ovis* larvae (L1). Indeed, the macroscopic examination revealed larvae of white color and about 1 mm length. Microscopically, these larvae were composed of eleven metameres, each of these displaying 4 rows of spines (Fig. 1a). The cephalic segment had two large black buccal hooks (Fig. 1b), while the posterior segment consisted of two tubercles, each containing about ten curved spines (Fig. 1c) which is concordant with the morphological description of L1 *Oestrus ovis* larvae in the literature [3].

At the first visit, the patient received a local treatment based on the administration of oxybuprocaine and antiseptics (Biocidan®) as eye drops. The curative treatment consisted of the mechanical removal of all of the eight larvae present at the level of the conjunctiva using a forceps, as mentioned before. Subsequently, the treatment was supplemented by the administration of antiseptic eye drops (i.e. desomedine) and antibiotics (i.e. ofloxacin). Removal of the larvae resulted in rapid relief and no complication was further reported.

## Discussion and conclusion

We described herein a case of human OM in a patient living within an area of farms when he first started displaying symptoms. This observation is in line with the classical epidemiological distribution of human OM reporting it as a rare cosmopolitan disease, usually observed in rural areas where *Oestrus ovis’* larvae usually parasitize the cavities of the frontal sinuses of sheep, causing sheep oestrosis. *Oestrus ovis’* larvae can also parasitize other species of domestic quadrupeds (e.g. goats, horses) and even, accidentally, humans [4]. Clinically, conjunctival myiasis manifests as (i) superficial external forms (EOM) classically associated with irritation, photophobia and pain, or (ii) deep internal forms (IOM) potentially associated with blindness. In the case report here, the clinical signs were in accordance to conventionally described signs of EOM as reported in the literature (Table 1). The treatment of human oestrosis is based on the extraction of the larvae. For EOM, local antibiotics can be combined to avoid superinfection [4]. In our case the complete cure took place after extraction of all of the larvae supplemented by the administration of antiseptic eye drops (i.e. desomedine) and antibiotics (i.e. ofloxacin).

**Table 1:** Case of external ophtalmomyiasis reported in France (Excluding Corsica) between 1918 and 2017 (*: unspecified or data unavailable)

Number of cases	Years	Age (years)	Sex	Profession	Complaint	Season / Month	Number of larvae	References
1	1959	13	*	*	*	*	8	[4]
11	1918–1952	6 to 42	*	*	*	*	1 to 15	[4]
1	1976	*	*	*	*	*	*	[4]
8	2000	11	M	School boy	Foreign body sensation	*	10	[7]
	1999	*	*	*	burning sensation	June	*	[5]
	1980	18	*	*	*	Summer	10	[8]
	2013	45	F	Teacher	Itching, irritation	June	*	[6]
	2013	67	F	Farmer	Painfull, Inflammation	July	*	
	2013	43	F	Nurse	Foreign body sensation	July	*	
	2013	28	M	Mason	Pain	July	*	
	1969	*	*	*	*	*	12	[4]

*Oestridae* are one of the families of Diptera most often involved in human OM. Three subfamilies of *Oestridae* may be involved in EOM (i) *Oestrinae* (i.e. *Oestrus ovis, Rhinoestrus purpureaous, Pharyngomyia picta, Cephenemyia ulrichii* and *Gedolstia häsleri)*, (ii) *Gasterophilinae* (i.e. *Gasterophilus intestinalis*) and (iii) *Hypodermatinae* (i.e. *Dermatobia hominis*) [1]. The differential diagnosis is based on the study of the morphology of the first- and/or the third-instar larva, particularly by studying the cephalic and the last segment and by chetotaxy. In our case, there was no particular difficulty to identify L1 larvae of *Oestrus ovis* (Fig. 1) [8].

*Oestrus ovis* is an insect frequently found in rural areas of tropical/subtropical and temperate regions (i.e. Europe, North America, New Zealand, Australia) [1]. Consequently, EOMs are predominantly found in the regions of North Africa, Middle East and South Asia [1]. Indeed, among 295 cases described worldwide between 1918 and 2017, 110 (37%) occurred in North Africa, 57 (20%) in Middle East and 31 (10%) in South Asia (Table 1). In Europe, EOMs are endemic in the Mediterranean Bassin with sporadic cases in central Europe. Thus, most of EOMs in France have been observed in the southern regions [4–8]. Moreover, due to climatic conditions and the presence of numerous sheep farms, Corsica is an ideal geographical area for the development of *Oestrus ovis,* explaining that most of the cases of EOMs in France have been identified in this Island [12]. Moreover, EOMs are mainly reported during the summer and early autumn period in hot regions, particularly near sheep or goat farming areas [4]. However, the number of cases is probably underestimated and the information is partial in the majority of the cases documented who do not specify the diagnostic season (Table 1).

The climate of the Mediterranean region is known to be propitious for the development of the biological cycle of numerous oestrids. Thus, since climatic conditions are not similar between the different European countries, the life cycle of *Oestrus ovis* is not similar between these different regions. For example, in 2010, Papadopoulos et al highlighted two annual peaks in adult flies activity in spring and late summer in Greece. Moreover, they observed that the spring peak occurred later and was less pronounced in goat than in sheep [13]. In Southern France, three generation of *Oestrus ovis* are usually observed along the year, leading to three annual peaks in spring, summer and autumn [13, 14]. The third peak observed in autumn in Southern France corresponds to a phase of hypobiosis during which *Oestrus ovis’* larvae development stop, explaining that animals are infected exclusively by larvae of the stage L1. Thus, from October to December, all larvae seen in southwestern France are L1 [15]. As a consequence, it could explain that only larvae L1 were found in our case. In addition, less prevalence of ectoparasite in livestock is observed in northerly Europe, although some cases of oestrosis have been described in UK [14, 15].

Thus, a case of EOM in the last week of September in a patient living in Burgundy that has no history of travel neither abroad nor in the south of France during the previous months is surprising, raising the question of the consequences of the recent global warming on insect ecological distribution throughout France. Indeed, a retrospective analysis of the temperature changes observed in Burgundy between 1961 and 2011 revealed a faster warming in the Burgundy region compared to the world average (+ 1 °C vs + 0.5 °C) [16]. This warming is more marked on diurnal temperatures, indicating a change in atmospheric humidity and/or cloud cover. Finally, this study demonstrated a more marked climatic warming during the vegetative season (spring and summer) in Burgundy [16]. Studies aimed at evaluating the impact of warming in the implantation of Diptera of the genus *Oestrus* sp. in Burgundy would help in (i) precising the extension of new ecological niches for Diptera to northern areas and (ii) appreciating the risk for EOM in formerly non-endemic areas.

We report here a case of EOM in the region of Burgundy (France) in the last week of September 2016. Although underestimated in humans, EOMs remains rare disorders with less than 300 cases recorded in the world in the last century. The majority of EOMs is classically observed in hot and dry regions of North Africa, the Middle East and South Asia. Finally, the diagnosis of an EOM in Burgundy, a French region described as cold and humid, is surprising and could be due to a more marked climatic warming during the vegetative season in Burgundy resulting in the implantation of Diptera of the genus *Oestrus* sp. in this region.

## Additional file

Additional file 1: **Figure S1.** Parasite cycle of *Oestrus ovis. Oestrus ovis* exerts a strict parasitism of the nasal cavities of small sheep and goat ruminants. The viviparous females of *Oestrus ovis* deposit first-stage larvae (L1) directly in the nasal orifices of sheep and goats. L1 actively penetrate through the nasal orifices and colonize the cornets and septum where they will develop. Once located at the ethmoid level, L1 molt to stage 2 larvae (L2). L2 further ascend from the nasal cavity to the frontal sinuses where they molt to stage 3 larvae (L3). Thereafter, L3 are expelled from the nasal cavity of the host by sneezing via the nasal mucus that subsequently contaminate the soils. Then, L3 turn into a pupa in 12–24 h. Finally, when the external conditions are favorable, the pupa molt into an adult fly in 30 to 34 days. Accidentally, L1 larvae can be deposited on or into the ocular cavities of human. (L1: Stage 1 larvae; L2: Stage 2 larvae; L3: Stage 3 larvae). (TIF 17963 kb)

## Abbreviations

EOM: External ophtalmomyiasis
IOM: Internal ophtalmomyiasis
OM: Ophtalmomyiasis
