# Supplementary figures and images for: Oestrus ovis external ophtalmomyiasis: a case report in Burgundy France
Source: BMC Ophthalmol. 2018 Dec 22;18:335. doi: 10.1186/s12886-018-1003-z (PMC6303972; doi:10.1186/s12886-018-1003-z)

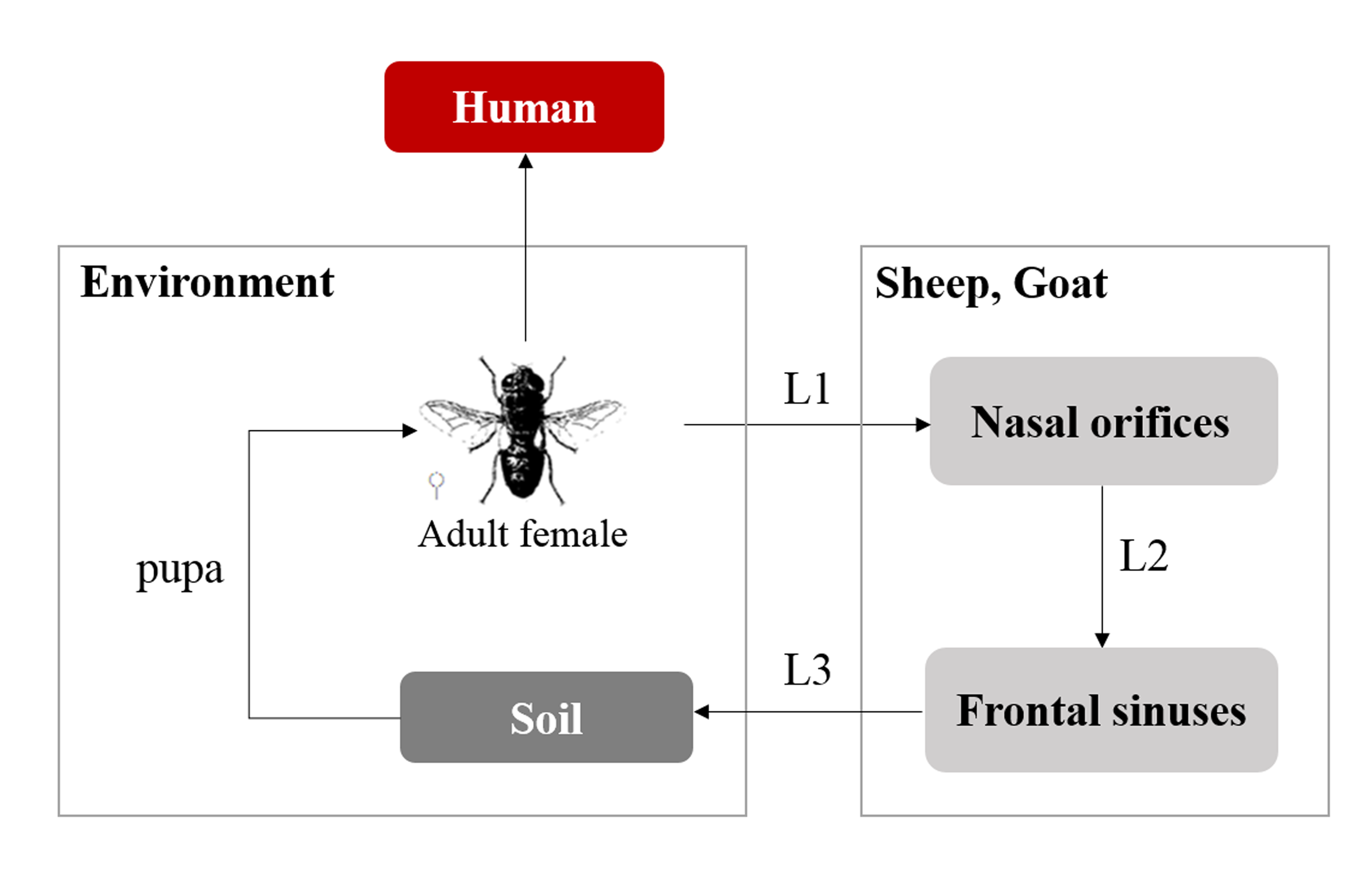

Supplement: Supplementary file 1 — Figure S1. Parasite cycle of Oestrus ovis. Oestrus ovis exerts a strict parasitism of the nasal cavities of small sheep and goat ruminants. The viviparous females of Oestrus ovis deposit first-stage larvae (L1) directly in the nasal orifices of sheep and goats. L1 actively penetrate through the nasal orifices and colonize the cornets and septum where they will develop. Once located at the ethmoid level, L1 molt to stage 2 larvae (L2). L2 further ascend from the nasal cavity to the frontal sinuses where they molt to stage 3 larvae (L3). Thereafter, L3 are expelled from the nasal cavity of the host by sneezing via the nasal mucus that subsequently contaminate the soils. Then, L3 turn into a pupa in 12–24 h. Finally, when the external conditions are favorable, the pupa molt into an adult fly in 30 to 34 days. Accidentally, L1 larvae can be deposited on or into the ocular cavities of human. (L1: Stage 1 larvae; L2: Stage 2 larvae; L3: Stage 3 larvae). (TIF 17963 kb) [file 12886_2018_1003_MOESM1_ESM.tif]
